# Chronic exposure to low doses of lipopolysaccharide and high-fat feeding increases body mass without affecting glucose tolerance in female rats

**DOI:** 10.14814/phy2.12584

**Published:** 2015-11-04

**Authors:** Anete Dudele, Christina W Fischer, Betina Elfving, Gregers Wegener, Tobias Wang, Sten Lund

**Affiliations:** 1 Section for Zoophysiology, Department of Bioscience, Aarhus UniversityAarhus, Denmark; 2 Translational Neuropsychiatry Unit, Department of Clinical Medicine, Aarhus UniversityRisskov, Denmark; 3 Medical Research Laboratory, Department of Endocrinology and Internal Medicine, Aarhus University HospitalAarhus, Denmark

**Keywords:** Chronic endotoxemia, female rats, glucose tolerance, high-fat diet

## Abstract

Obesity-related inflammation may have a causal role in the development of diabetes and insulin resistance, and studies using animal models of chronic experimental endotoxemia have shown the link. However, many studies use only males, and much less is known about the role of obesity-related inflammation in females. Therefore, we addressed how experimentally induced chronic inflammation affects body mass, energy intake, and glucose metabolism in female rats. Adult female Sprague Dawley rats were instrumented with slow release pellets that delivered a constant daily dose of 53 or 207 *μ*g of lipopolysaccharide (LPS) per rat for 60 days. Control rats were instrumented with vehicle pellets. Due to inflammatory nature of high-fat diet (HFD) half of the rats received HFD (60% of calories from lard), while the other half remained on control diet to detect possible interactions between two modes of induced inflammation. Our results showed that chronic LPS administration increased female rat body mass and calorie intake in a dose-dependent manner, and that HFD further exacerbated these effects. Despite these effects, no effects of LPS and HFD were evident on female rat glucose metabolism. Only LPS elevated expression of inflammatory markers in the hypothalamus. To conclude, female rats respond to experimentally induced chronic inflammation by increasing body mass, but do not develop glucose intolerance in the given period of time.

## Introduction

Many epidemiological studies implicate the immune system as the culprit in obesity-related diseases, showing associations between obesity, type 2 diabetes (T2D), and cardiovascular disease with elevated levels of inflammatory markers in the blood (Koenig et al. 1999; Pradhan et al. 2001; Park et al. 2005). The causal relationship between low-grade inflammation and such lifestyle diseases as insulin resistance, coronary heart disease, and atherosclerosis is reinforced from experimental induction of inflammation in animal models (Hotamisligil et al. 1993; Cani et al. 2007; Kleemann et al. 2008; Smith et al. 2009). Although the exact origin of inflammation during obesity remains debated, consumption of high-fat diet (HFD) can initiate and promote systemic inflammation in several ways, particularly via induction of endotoxemia – elevation of lipopolysaccharide (LPS) in the circulation (reviewed in [Kelly et al. 2012]). LPS is a potent endotoxin that naturally occurs on the outer surface of the Gram-negative bacteria in intestinal lumen and activates the innate immune response via Toll-like receptor 4 (TLR4) and Cluster differentiation 14 (CD14)-dependent pathways (Alexander and Rietschel 2001; Lu et al. 2008). HFD can contribute to endotoxemia by altering the composition of intestinal microbiome to raise bacterial LPS production or by increasing intestinal permeability such that more LPS is absorbed across the intestinal wall into the circulation (Cani et al. 2008; Laugerette et al. 2011; Kelly et al. 2012). Furthermore, dietary fatty acids per se may activate the innate immune response via the TLR4 pathway (Shi et al. 2006). Activation of the innate immune response by LPS and fatty acids can then interfere with insulin-dependent glucose uptake leading to development of insulin resistance (de Luca and Olefsky 2008), and consumption of HFD can initiate inflammation in the hypothalamus altering hypothalamic sensitivity to insulin and leptin and affecting appetite regulation (De Souza et al. 2005; Thaler et al. 2012). Hence, the immune system is intimately involved in the regulation of energy homeostasis and metabolism.

So far, chronic experimental elevation of LPS has been shown to induce obesity in mice, insulin resistance in humans and mice (Cani et al. 2007; Amar et al. 2008), and cardiovascular pathologies in rats (Smith et al. 2009). However, these effects of LPS have been investigated in males (Cani et al. 2007; Amar et al. 2008; Smith et al. 2009), and much less is known about the responses in females (Pohl et al. 2013). Gender and sex hormones undeniably have a crucial role in regulation of metabolism and may affect inflammatory reactions and susceptibility to the development of T2D (Pietschmann et al. 2003; Logue et al. 2011). Therefore, it is important to investigate the effects of chronic inflammation on female physiology, focusing on glucose handling and adiposity.

We hypothesized that a low-level chronic inflammation, induced by continuous LPS delivery, will lead to weight gain and insulin resistance in female Sprague Dawley rats. LPS was delivered into the peritoneal cavity via implantation of slow release pellets lasting for 60 days to mimic endotoxemia that arises from increased LPS absorption from the intestine. Furthermore, we wished to investigate whether the effects of LPS are exacerbated by HFD, due to its inflammatory nature and previously observed alteration of response to LPS in obese animals (Shi et al. 2006; Lawrence et al. 2012).

## Materials and Methods

### General procedures

Sprague Dawley female rats (*n* = 34) were obtained from Taconic (Lille Skensved, Denmark) at the age of 8 weeks. They were housed in groups of 2–3 with a 12:12-hour light:dark cycle with lights on at 0600, a relative humidity of 45–70%, and an ambient temperature of 23°C. The rats always had ad libitum access to food and water, except for the overnight fast preceding the oral glucose tolerance test (OGTT), where food was removed 16 h prior to the test.

**Figure 1:**
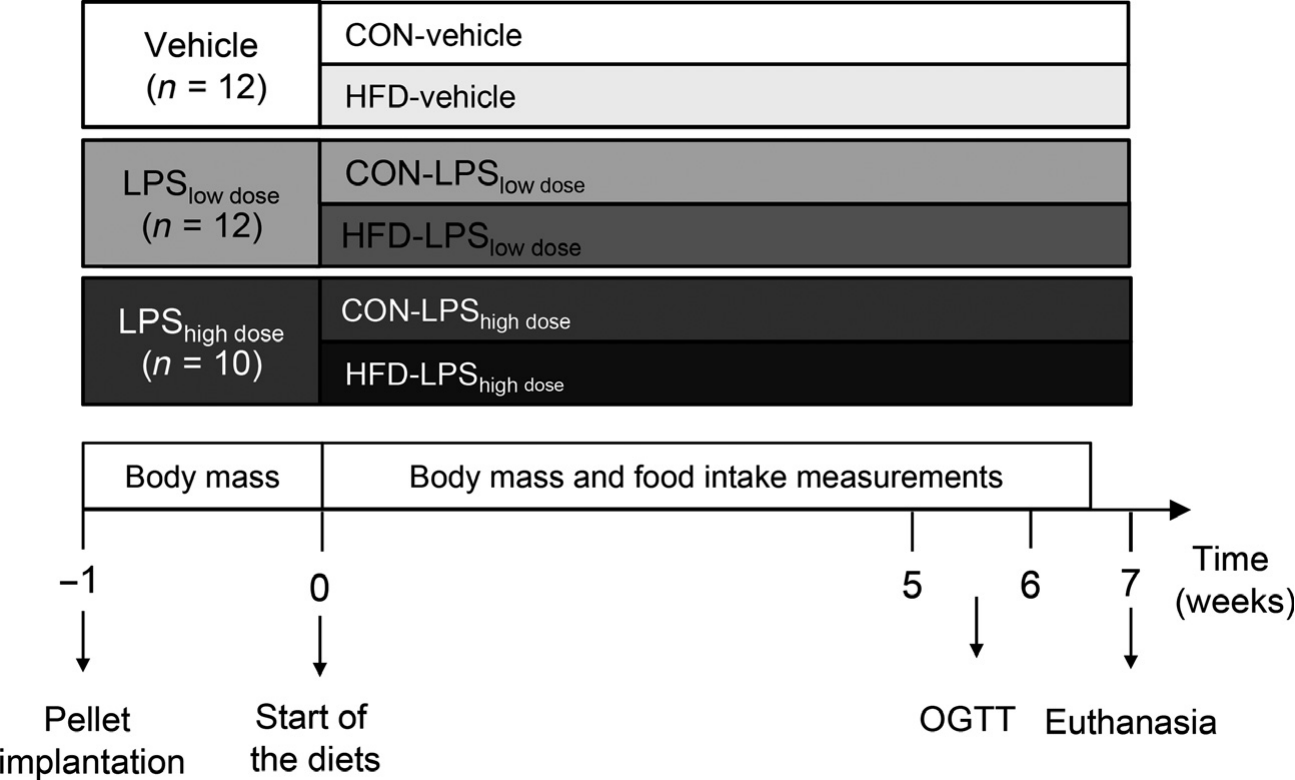
Experimental protocol. Thirty-four female Sprague Dawley rats were implanted with an intraperitoneal slow release pellet continuously delivering either a low (53 *μ*g·day^−1^) or a high (207 *μ*g·day^−1^) dose of lipopolysaccharide (LPS), or containing only pellet matrix. One week after the surgery, half of the rats were switched onto a high-fat diet (HFD), while the other half were given a control (CON) diet. Body mass and food intake was measured for 6.5 weeks after diet change. Oral glucose tolerance test (OGTT) was performed 6–7 weeks after pellet implantation. Rats were euthanized 8 weeks after pellet implantation.

After 1 week of acclimation, rats were weighed and divided into six treatment groups (Fig.1). As outlined below, they were equipped with a custom-made time release pellet (Innovative Research of America, Sarasota, FL) designed to deliver either a low dose (53 *μ*g·day^−1^, *n* = 12) or a high dose (207 *μ*g·day^−1^, *n* = 10) of LPS from *Escherichia coli* 055:B5 (Sigma-Aldrich, St. Louis, MO) at a constant rate for 60 days. The remaining 12 rats were implanted with the pellet matrix for control treatment (Vehicle). A week after the operation, half of the animals in each group were switched to a high-fat diet (HFD; #D12492, 60% kcal from fat), while the other half continued on the normal low fat, control diet (CON; #D12450B, 10% kcal from fat). Both diets were produced by Research Diets, Inc. (New Brunswick, NJ), and purchased from Brogaarden (Gentofte, Denmark). The D12450B diet was chosen as it is a recommended control diet by Research Diets, Inc. and is commonly used as a control diet for D12492. Even though other diets may present a better match for D#12492 in terms of sucrose levels, previous studies have shown development of obesity, inflammation, and insulin resistance when using D12450B as a control diet for a high-fat diet (Ghibaudi et al. 2002; Posey et al. 2009).

Body mass was measured from the day of pellet implantation and food intake was measured from the start of the diets. Change in body mass was calculated as the difference between measured body mass and body mass before pellet implantation.

OGTT was performed 6–7 weeks after pellet implantation (see OGTT) and rats were killed 8 weeks after pellet implantation (Fig.1).

All experiments were performed in strict accordance with the protocols specifically approved by The Danish Animal Experiments Inspectorate, permit # 2012-15-293400042.

### Pellet implantation

Rats were anesthetized by a subcutaneous injection of fentanyl/fluanisone and midazolam (2.5 mg·kg^−1^and 1.25 mg·kg^−1^, respectively), placed on a heating mat, and given a subcutaneous dose of Rimadyl® (Carprofen, 5 mg·kg^−1^). The incision area was shaved and disinfected before a 0.5- to 1-cm long midline incision gained access to the peritoneal cavity, allowing insertion of the slow release pellet. The incision was sutured appropriately, and sprayed with a disinfectant agent. All surgical procedures were performed under sterile conditions. Rats were allowed to wake up under a heating lamp and were then returned to the stabling facility. They all received a daily subcutaneous injection of Rimadyl® (Carprofen, 5 mg·kg^−1^) for the first 2 days after the surgery to reduce pain and acute inflammatory response as result of the operation.

### Oral glucose tolerance test

Oral glucose tolerance tests were performed 6–7 weeks after pellet implantation. Rats were fasted overnight (approximately 16 h) and blood for measurements of fasting glucose and insulin was acquired through a small cut made by a sterile scalpel at the tip of the tail. It was followed by an oral gavage of glucose (2.5 g·kg^−1^). Blood glucose levels were measured at 15, 30, 60, and 120 min and plasma was collected at these time points for insulin measurements. Blood glucose was determined using One Touch Ultra® glucometer and test strips (Life Scan, Inc., Milpitas, CA). Plasma insulin was determined using an ultra-sensitive rat insulin ELISA kits (DRG Diagnostics GmbH, Marburg, Germany).

### Termination procedure

Eight weeks after pellet implantation, rats were lightly anesthetized with isoflurane, killed by cervical dislocation, and decapitated. Trunk blood was collected, plasma separated, and immediately frozen for later analyses. Hypothalamus was dissected immediately, snap frozen on dry ice, and stored at −80°C for later analysis of cytokines and appetite-regulating genes. Liver, pancreas, heart, and spleen were dissected for measurements of wet mass. Visceral fat depots surrounding the mesentery, the ovaries, and behind the kidneys were dissected and weighed.

### Quantification of cytokines and appetite-regulating genes by quantitative real-time polymerase chain reaction (real-time qPCR)

Gene expression of the proinflammatory cytokines interleukin-1*β* (IL-1*β*), tumor necrosis factor-alpha (TNF-*α*), IL-6, and appetite-regulating genes: agouti-related protein (AgRP), neuropeptide Y (NPY), NPY receptors 1 and 2 (NPYR1, NPYR2), proopiomelanocortin (POMC), orexin, promelanin-concentrating hormone (Pmch), leptin receptor (LR), the obesity gene (FTO), cocaine-and-amphetamine–regulated transcript (Cart) were determined with real-time qPCR (*n* = 5 per group). Tissue homogenization and RNA extraction were carried out as described previously (Elfving et al. 2008).

#### RNA characterization

The integrity of RNA and the RNA concentration was determined with RNA StdSens microfluidic chips using the Experion Automated Electrophoresis System (BIORAD, CA). The RNA purity and the RNA concentration were determined by a NanoDrop 1000 spectrophotometer (Thermo Fischer Scientific).

#### cDNA synthesis

Before cDNA synthesis, the RNA concentration of the samples was adjusted to match the sample with the lowest concentration determined by the NanoDrop spectrophotometer. RNA was reversely transcribed using qScript™ cDNA SuperMix (Quanta Biosciences™) following manufacturer’s instructions, with RNA concentrations of 36 ng *μ*L^−1^ per reaction. The cDNA samples were stored undiluted at −80°C until real-time qPCR analysis. All samples were diluted 1:14 with DEPC-treated water prior to real-time qPCR analysis.

#### Real-time qPCR

The real-time qPCR reactions were carried out in 96-well PCR plates using the Mx3000P (Stratagene) and SYBR Green. The gene expression of eight reference genes (18sRNA, ActB, CycA, Gapd, Hmbs, Hprt1, Rpl13A, and Ywhaz), three cytokines (IL-1*β*, IL-6, and TNF-*α*), and 10 genes related to appetite regulation (AgRP, NPY, NPYR1, NPYR2, POMC, orexin, Pmch, LR, FTO, and Cart) were investigated (Table1). The reference genes were selected as described by Bonefeld et al. (Bonefeld et al. 2008) and the primers were designed as described by Elfving et al. (Elfving et al. 2008). Each SYBR Green reaction (10 *μ*L total volume) contained 1xSYBR Green master mix (BIORAD, CA), 0.5 μmol/L primer pairs, and 3 *μ*L of diluted cDNA and were carried out as described previously (Elfving et al. 2008). All samples were run in duplicate, and a standard curve (performed in duplicate) was generated on each plate.

**Table 1:** Characteristics of gene-specific real-time qPCR primers

Gene symbol	Gene name	Accession no.*	Primer sequence	Amplicon size†
18s rRNA	18s subunit ribosomal RNA	M11188	(+) acggaccagagcgaaagcat(−) tgtcaatcctgtccgtgtcc	310
ActB	Beta-actin	NM_031144	(+) tgtcaccaactgggacgata(−) ggggtgttgaaggtctcaaa	165
CycA	Cyclophilin A	XM_345810	(+) agcactggggagaaaggatt(−) agccactcagtcttggcagt	248
Gapd	Glyceraldehyde-3-phosphate dehydrogenase	NM_017008	(+) tcaccaccatggagaaggc(−) gctaagcagttggtggtgca	168
Hmbs	Hydroxymethylbilane synthase	NM_013168	(+) tcctggctttaccattggag(−) tgaattccaggtgagggaac	176
Hprt1	Hypoxanthine guanine phosphoribosyl transferase 1	NM_012583	(+) gcagactttgctttccttgg(−) cgagaggtccttttcaccag	81
Rpl13A	Ribosomal protein L13A	NM_173340	(+) acaagaaaaagcggatggtg(−) ttccggtaatggatctttgc	167
Ywhaz	Tyrosine-3-monooxygenase/tryptophan-5-monooxygenase activation protein, zeta	BC094305	(+) ttgagcagaagacggaaggt(−) gaagcattggggatcaagaa	136
IL-1β	Interleukin1beta	NM_031512.2	(+) tgcccgtggagcttccagga(−) tccagctgcagggtgggtgt	359
IL-6	Interleukin 6	NM_012589.2	(+) tctgtctcgagcccaccaggaa(−) ctggctggaagtctcttgcgga	91
TNF-α	Tumor necrosis factor-alpha	NM_012675.3	(+) gaccctcacactcagatcatcttct(−) acgctggctcagccactc	106
AgRP	Agouti-related protein	NM_033650.1	(+) ttcccagagttctcaggtctaagt(−) atctagcacctctgccaaagc	99
NPY	Neuropeptide Y	NM_012614.2	(+) tggactgaccctcgc(−) tgtctcagggctggatctct	187
NPYR1	NPY receptor 1	NM_001113357.1	(+) gtctcccgttcacctttgta(−) ccaccagcgatgagaaccaga	132
NPYR2	NPY receptor 2	NM_023968.1	(+) tgtgcctgccattcactctt(−) gcaacgatgtcggtccaaag	149
POMC	Proopiomelanocortin	NM_139326.2	(+) caggacctcaccacggaaag(−) tgacgtacttccggggattt	114
Pmch	Promelanin-concentrating hormone	NM_012625.1	(+) cattcaggatggggaaagcc(−) gcctgtgttctttgtggtct	137
Orexin	Orexin	NM_013179.2	(+) ttctacaaaggttccctgggc(−) gtagagacggcaggaacacg	136
LR	Leptin receptor	NM_012596.1	(+) tgggtttgcgtatggaagtca(−) acgatttcagcagcctctct	139
FTO	The obesity gene	NM_001039713.1	(+) gagttctatcagcagtggcag(−) ctcaccaggtcccgaaacaa	143
Cart	Cocaine-and-amphetamine–regulated transcript	NM_017110.1	(+) ggcgctgtgttgcagattga(−) agcgtcacacatggggactt	108

* Genbank accession number of cDNA and corresponding gene, available at http://www.ncbi.nlm.nih.gov/.

† Amplicon length in base pairs.

Gene-specific data on primer sequence and amplicon sizes are given in Table1. Primers were obtained from Sigma–Aldrich, Denmark.

Data normalization was carried out based on the best combination of reference genes mRNA expression, using Normfinder software (http://www.mdl.dk) (Andersen et al. 2004). Values for each individual gene were normalized with the geometric mean of the reference genes Hprt and Gapd. Data are presented as percentage of CON-vehicle ± standard error of mean (SEM).

### Data analysis

Body mass during the first week was analyzed using repeated measures analysis of variance (ANOVA), with Tukey’s test for multiple comparison. Continuous body mass and food intake were analyzed using two-way repeated measures (RM) ANOVA. Total food and calorie intake, and weight gain 6.5 weeks after the start of the experiment, calculated in relation to body mass before pellet implantation, area under the curve (AUC) for glucose and insulin levels, organ weight, and gene expression were analyzed using two-way ANOVA, followed by Tukey’s test for multiple comparison. Organ mass was analyzed using two-way ANOVA and two-way analysis of covariance (ANCOVA) with body mass as the covariate.

All statistical analyses were performed using Prism (version 6.0e PRISM 6 for MAC OS X), with an alpha level of 5%. All data are presented as means ± SEM.

## Results

### Body mass and food and energy intake

**Figure 2:**
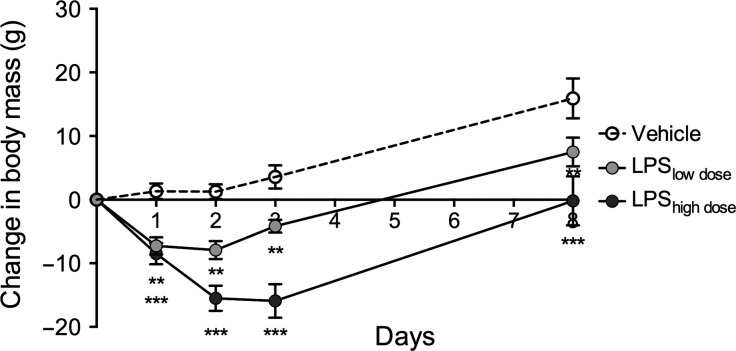
Body mass after pellet implantation. LPS significantly reduced body mass during the first 8 days after pellet implantation in a dose-dependent manner (*F*_8,124_ = 5.45, *P* < 0.0001). Data are presented as means ± SEM (*n* = 5–6 rats per group). * indicates significant effect of LPS vs. Vehicle with **P < 0.01,***P < 0.001 (Tukey’s test).

Body mass was similar in the groups at the beginning of the experiment (*F*_2,31_ = 1.35, *P* = 0.27) with rats weighting an average of 258 ± 3 g. During the first 8 days, there was a significant LPS and time interaction on body mass (*F*_8,124_ = 5.45, *P* < 0.0001). Rats exposed to LPS_low dose_ and LPS_high dose_ lost body mass during the first 3 days following surgery compared to vehicle rats and retained lower body mass until day 8 (Fig.2).

**Figure 3:**
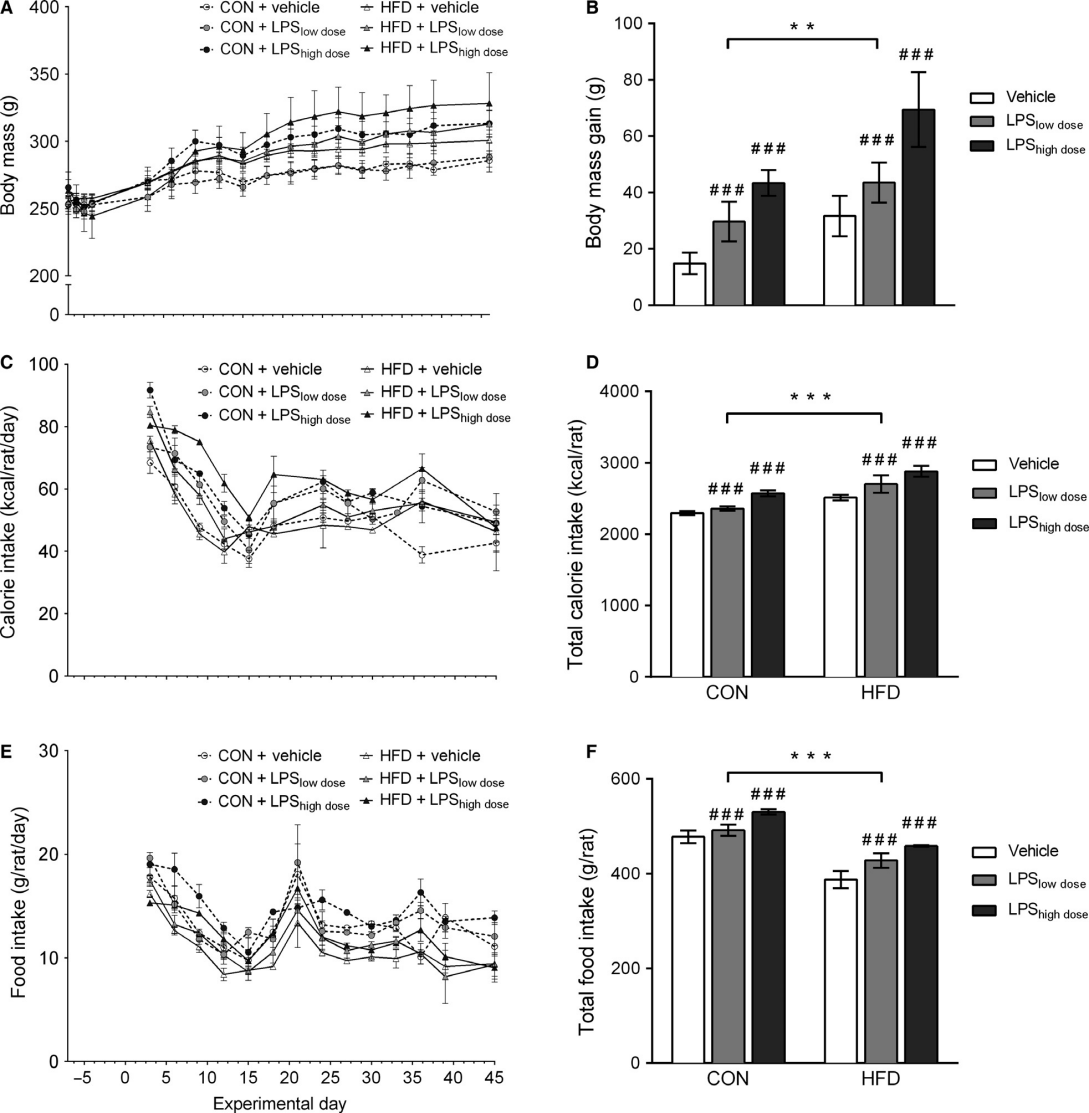
Body mass and energy and food intake. (A) Body mass throughout the experiment (RM ANOVA, HFD: *F*_1,28_ = 3.09, *P* = 0.09; LPS: *F*_2,28_ = 2.88, *P* = 0.07). (B) Total body mass gain 6.5 weeks after the start of the experiment (two-way ANOVA, HFD: *F*_1,28_ = 9.46, *P* = 0.005; LPS: *F*_2,28_ = 9.46, *P* = 0.0007). (C) Daily calorie intake (RM two-way ANOVA, HFD: *F*_1,28_ = 54.07, *P* < 0.0001; LPS: *F*_2,28_ = 21.75, *P* < 0.0001). (D) Total energy intake at the end of the experiment (two-way ANOVA, HFD: *F*_1,28_ = 28.93, *P* < 0.0001; LPS: *F*_2,28_ = 11.63, *P* = 0.0002). (E) Daily food intake (two-way RM ANOVA, HFD: *F*_1,28_ = 2.87, *P* < 0.0001; LPS: *F*_2,28_ = 1.74, *P* < 0.0001). (F) Total food intake (two-way ANOVA, HFD: *F*_1,28_ = 102.7, *P* < 0.0001; LPS: *F*_2,28_ = 17.25, *P* < 0.0001). Data are presented as means ± SEM (*n* = 5–6 rats per group). ^#^ indicates significant effect of LPS, with ^###^*P* < 0.001, and * indicates significant effect of HFD, with ***P < 0.001.

LPS and HFD showed a nonsignificant tendency to increase absolute body mass measured throughout the experiment (Fig.3A) nevertheless total body mass gain 6.5 weeks after the start of the experiment was significantly increased by HFD and LPS (Fig.3B).

Daily food and energy intake was markedly affected by LPS and HFD (Fig.3C, E). While total food intake was increased by LPS treatment, it was reduced by HFD exposure (Fig.3F). Total energy intake (6.5 weeks), on the other hand, was elevated by both HFD and LPS (Fig.3D, F).

### Oral glucose tolerance test (OGTT)

**Figure 4:**
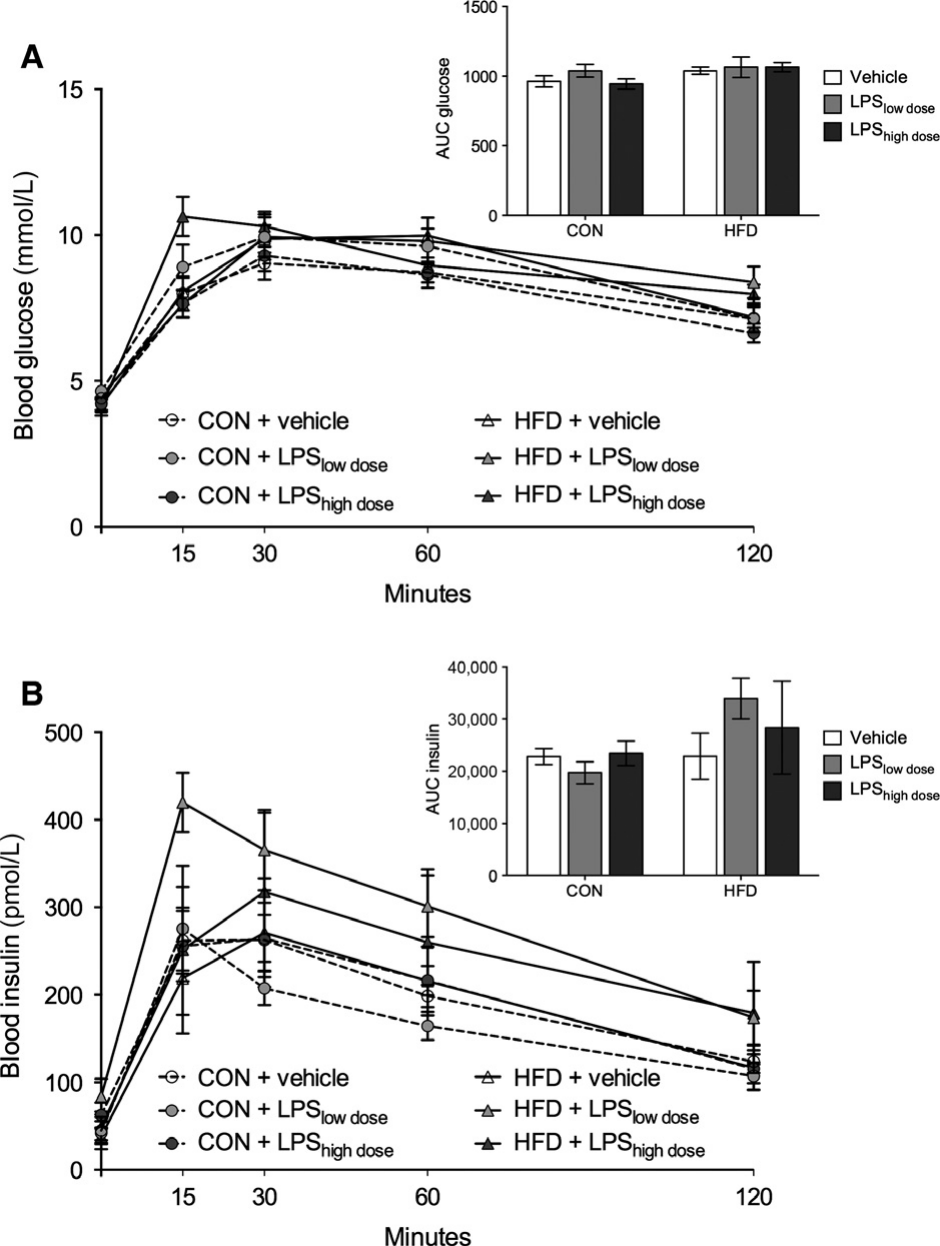
Oral glucose tolerance test (OGTT) 6–7 weeks after pellet implantation. (A) Blood glucose and (B) insulin levels during OGTT and calculated area under the curve (AUC) for glucose and insulin. AUC_glucose_ (two-way ANOVA, HFD: *F*_1,28_ = 3.85 *P* = 0.06; LPS: *F*_2,28_ = 0.79, *P* = 0.47), AUC_insulin_ (two-way ANOVA, HFD: *F*_1,28_ = 3.26, *P* = 0.08, LPS: *F*_2,28_ = 0.49, *P* = 0.62). Data are presented as means ± SEM (*n* = 5–6 rats per group).

There was a tendency for HFD to increase plasma glucose and insulin levels during an OGTT, assessed by calculating the AUC, but there was no effect of LPS on either plasma insulin or glucose (Fig.4).

### Organ mass

Visceral fat was significantly increased by HFD (two-way ANOVA, *F*_1,28_ = 11.68, *P* = 0.002), but not by LPS (two-way ANOVA: *F*_2,28_ = 1.24, *P* = 0.31; Table2). However, these differences disappeared when body mass was included in the analysis (two-way ANCOVA, HFD: *F* = 3.81, *P* = 0.06; LPS: *F* = 1.89, *P* = 0.17; body mass: *F* = 13.41, *P* < 0.001). Similarly, mesentery fat was significantly increased by HFD (*F*_1,24_ = 6.58, *P* = 0.017), but there was no effect of LPS (*F*_2,24_ = 2.57, *P* = 0.10). After including body mass in the analysis, HFD lost its effect (two-way ANCOVA, *F* = 0.30, *P* = 0.59), while LPS proved to have a significant effect (two-way ANCOVA, LPS: *F* = 3.91, *P* = 0.03; body mass: *F* = 34.59, *P* < 0.0001).

**Table 2:** Organ weight

	Vehicle			High-fat diet		
	Vehicle	LPS_low dose_	LPS_high dose_	Vehicle	LPS_low dose_	LPS_high dose_
Visceral fat (g)*	17.51 ± 0.73	14.26 ± 0.92	16.92 ± 1.41	22.02 ± 1.98	20.10 ± 1.96	21.13 ± 2.11
Mesentery fat (g)*,‡	3.86 ± 0.21	2.78 ± 0.13	3.51 ± 0.49	4.55 ± 0.48	3.73 ± 0.41	5.00 ± 0.89
Spleen (g)†,‡	0.64 ± 0.04	0.80 ± 0.07	0.92 ± 0.07	0.74 ± 0.05	0.95 ± 0.07	0.95 ± 0.05
Liver weight (g)	9.27 ± 0.36	8.88 ± 0.30	9.90 ± 0.39	8.87 ± 0.56	10.23 ± 0.49	10.56 ± 0.66
Heart (g)	1.07 ± 0.04	1.01 ± 0.04	1.08 ± 0.01	1.01 ± 0.03	1.09 ± 0.05	1.12 ± 0.06
Pancreas (g)	1.18 ± 0.10	1.30 ± 0.12	1.38 ± 0.05	1.24 ± 0.16	1.36 ± 0.09	1.59 ± 0.15

Data are presented as means ± SEM (*n* = 5–6 per group).

* Significant effect HFD (high-fat diet, two-way ANOVA, *P* < 0.05).

† Significant effect of LPS (two-way ANOVA, *P* < 0.05).

‡ Significant effect of LPS (two-way ANCOVA, body mass included as a covariate, *P* < 0.05).

Spleen was markedly enlarged by LPS (two-way ANOVA, *F*_2,28_ = 9.06, *P* = 0.0009) with the effects persisting after correction for body mass (two-way ANCOVA, *F* = 8.88, *P* = 0.001), while there was only a trend toward an increased spleen mass by HFD (*F*_1,28_ = 3.74, *P* = 0.06) that disappeared after correction for body mass (two-way ANCOVA, HFD: *F* = 0.43, *P* = 0.52; body mass: *F* = 7.77, *P* = 0.009). Absolute, liver mass was not affected by LPS (F_2,28_=2.92, *P* = 0.07) or HFD (*F*_1,28_ = 1.94, *P* = 0.17), and correction for body mass did not reveal additional effects (two-way ANCOVA, HFD: *F* = 0.8, *P* = 0.38; LPS: *F* = 1.76, *P* = 0.19; body mass: *F* = 27.02, *P* < 0.0001).

Neither HFD nor LPS affected cardiac mass (HFD: *F*_1,28_ = 0.23, *P* = 0.64; LPS: *F*_2,28_ = 0.90, *P* = 0.43) and pancreatic mass (HFD: *F*_1,28_ = 1.27, *P* = 0.27; LPS: *F*_2,28_ = 2.70, *P* = 0.09), but both were affected by body mass (two-way ANCOVA, cardiac mass, HFD: *F* = 3.06, *P* = 0.09; LPS: *F* = 0.35, *P* = 0.71; body mass: *F* = 22.35, *P* < 0.0001; pancreatic mass, HFD: *F* = 0.38, *P* = 0.54; LPS: *F* = 1.58, *P* = 0.22; body mass *F* = 13.48, *P* = 0.001).

### Gene expression in the hypothalamus

**Figure 5:**
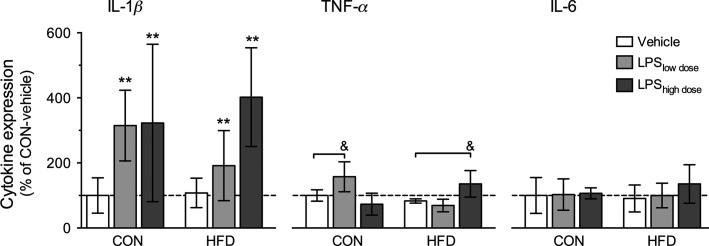
Proinflammatory cytokine expression in hypothalamus. IL-1*β* (two-way ANOVA, HFD: *F*_1,22_ = 0.09, *P* = 0.77, LPS: *F*_2,22_ = 6.00, *P* = 0.008), IL-6 (two-way ANOVA, HFD: *F*_1,22_ = 0.08, *P* = 0.79; LPS: *F*_2,22_ = 0.54, *P* = 0.59), TNF-*α* (two-way ANOVA, interaction between LPS × HFD: *F*_2,22_ = 14.57, *P* = 0.0001; Tukey test, CON-LPS_low dose_ versus CON-vehicle and HFD-LPS_high dose_ versus HFD-vehicle, *P* < 0.05). Data are presented as mean percentage of CON-vehicle ± SEM (*n* = 4–5 rats per group). * indicates significant effect of LPS, with **P < 0.01 (two-way ANOVA), & indicates significant difference from control, with ^&^*P* < 0.05 (Tukey’s test).

The mRNA expression of the cytokines and the appetite-regulating genes are given in Figure5 and Table3.

**Table 3:** Appetite-regulating gene expression in hypothalamus

Gene	Control diet			High-fat diet		
	Vehicle	LPS_low dose_	LPS_high dose_	Vehicle	LPS_low dose_	LPS_high dose_
Orexin*	100.0 ± 18.16	171.6 ± 25.66	72.27 ± 20.59	93.03 ± 23.93	154.8 ± 26.05	84.63 ± 16.09
AgRP	100.0 ± 20.44	85.51 ± 10.52	91.54 ± 20.93	76.02 ± 6.87	97.97 ± 16.67	95.16 ± 15.76
NPY	100.0 ± 19.57	94.14 ± 9.23	63.25 ± 1.60	74.15 ± 6.91	84.38 ± 5.11	85.12 ± 14.97
NPYR1	100.0 ± 8.21	81.03 ± 3.80	93.26 ± 10.04	96.04 ± 6.11	94.76 ± 4.84	104.8 ± 6.47
NPYR2	100.0 ± 19.47	103.8 ± 7.89	101.7 ± 6.64	118.2 ± 3.36	116.7 ± 5.09	104.8 ± 5.21
POMC	100.0 ± 15.16	86.38 ± 7.10	73.41 ± 8.43	77.25 ± 13.37	84.07 ± 7.49	78.70 ± 6.68
Pmch	100.0 ± 19.45	134.8 ± 9.48	141.7 ± 6.79	134.3 ± 14.94	131.8 ± 10.73	113.6 ± 21.77
LR	100.0 ± 10.20	112.7 ± 18.96	78.21 ± 8.71	83.36 ± 16.36	81.00 ± 8.81	96.05 ± 19.66
FTO	100.0 ± 3.47	92.53 ± 5.76	89.68 ± 8.82	88.15 ± 4.24	96.82 ± 2.88	92.05 ± 7.43
Cart	100.0 ± 6.72	93.25 ± 6.40	91.99 ± 9.03	99.50 ± 11.28	97.41 ± 5.07	111.1 ± 13.84

Data are presented as mean percentage of CON-vehicle ± SEM (*n* = 4–5 per group).

* Significant effect of LPS (two-way ANOVA, *P* < 0.05).

LPS significantly increased IL-1*β* expression, whereas there was no effect of HFD (Fig.5). There was an LPS and diet interaction on TNF-*α* expression: LPS_low dose_ significantly increased TNF-*α* expression in rats receiving CON diet, whereas LPS_high dose_ increased TNF-*α* in rats receiving HFD. IL-6 expression was not affected by LPS or HFD.

LPS had a significant effect on orexin expression (*F*_2,24_ = 4.61, *P* = 0.02), but no other changes in appetite-regulating gene expression were observed (Table3).

## Discussion

The present study showed that chronic exposure to relatively low doses of LPS increases body mass and food and calorie intake in adult female Sprague Dawley rats in a dose-dependent manner, but is not associated with impaired glucose tolerance. The LPS-induced effects on body mass and calorie intake were exacerbated by high-fat feeding, but only LPS-induced inflammation in the hypothalamus. HFD did not affect expression of appetite-regulating genes in the hypothalamus, and only orexin expression was affected by LPS.

Female rats exposed to LPS showed a significant and dose-dependent loss of body mass during the first 3 days after pellet implantation (Figs.2, 3A). This response was likely a part of the acute phase response characterized by anorexia, muscle cell catabolism, fever, and elevated levels of proinflammatory cytokines (Gruys et al. 2005). Thus, the dose-dependent body mass loss clearly showed that LPS pellets were functional and delivered two different doses of LPS from day 1 (Fig.2). Moreover, it showed that surgery and handling required for pellet implantation did not impose overt stress on the rats as sham-treated rats that were merely given the vehicle did not lose body mass after insertion of the pellets (Fig.2). One week after surgery all rats had recovered their body mass to the level of presurgical values or higher (Fig.2), a pattern observed previously (Lugarini et al. 2005), however rats receiving the high dose of LPS remained smaller, while rats undergoing sham surgery exhibited the largest weight gain. Even though LPS and HFD only showed a tendency for increasing the absolute body mass throughout the experiment (Fig.3A), the increase in body mass gain 6.5 weeks after the start of the experiment (7.5 weeks after pellet implantation) was markedly elevated by LPS and HFD (Fig.3B). LPS increased body mass in a dose-dependent manner, where rats receiving high dose of LPS had the largest body mass gain, those with low dose of LPS had lower weight gain, and rats receiving vehicle had the lowest weight gain (Fig.3B). High-fat feeding had an additive effect and further increased body mass gain among all experimental groups (Fig.3B). This is in accordance with a study in male mice where LPS-induced inflammation after 4 weeks of exposure increased body mass and fat depots (Cani et al. 2007), but studies investigating similar, low-dose chronic exposures to LPS in Sprague Dawley male rats have described opposite effects. Male rats exposed to a chronic LPS infusion for 10 days had lower body mass (O’Reilly et al. 1988) and body mass gain was reduced by exposure to LPS throughout 3 months of experiment (Smith et al. 2009). As LPS elicits different responses in body mass after acute (8 days, Fig.2) and chronic (7.5 weeks) exposure, it is important to identify the underlying mechanisms. It is also important to further investigate how chronic inflammation changes body composition, as here we observed an increase in body mass, but not in visceral fat deposition in response to LPS (Fig.3, Table2) and only HFD increased the size of visceral fat depots, but the effect disappeared when body mass was included as a covariate in the analysis (Table2). As we did not measure the size of subcutaneous fat depots, it is not possible to conclude whether chronic LPS infusion increased lean or adipose tissue mass.

Increase in body mass stems from energy intake exceeding energy expenditure (Hill 2006). As we did not measure metabolic rate, it is possible that energy expenditure decreased in response to inflammation or high-fat feeding, but it is more likely that body mass rose in response to increased appetite. In the rats receiving the high LPS dose and HFD, the group with the largest weight gain, the rise in body mass became evident from day 15, but a tendency for increased caloric intake could be observed already after day 6 (Fig.3A, C). While it is difficult to elucidate a clear pattern of changes in food or calorie intake over time and the corresponding changes in weight gain across all experimental groups, the overall observation of increased food and caloric intake and body mass in LPS-treated animals is rather intriguing. Upon acute exposure, LPS has anorexic effects (Geary et al. 2004), but much less is known about chronic effects and here we showed that chronic exposure to LPS increased both food and caloric intake of female rats in a dose-dependent manner. HFD, on the other hand, lowered absolute food intake (measured in grams per rat), but due to higher energy density (5.24 kcal·g^−1^ for HFD vs. 3.9 kcal·g^−1^ for control diet) resulted in increased total energy intake. This has previously been observed in rodents that have a limited capability to downregulate consumption of energy-dense diets, thus leading to caloric surplus (Hariri and Thibault 2010). While combination of LPS and HFD exposure further increased caloric intake and showed an additive effect, the differential effect of LPS and HFD on food intake may implicate involvement of divergent mechanisms involved in appetite regulation. One of the possibilities is hypothalamic inflammation, involved in early onset and development of obesity (Thaler et al. 2012). While previous reports showed that high-fat feeding can induce hypothalamic inflammation already after 3 days of exposure and persists after 4 and even 20 weeks in male rats (Thaler et al. 2012), we did not observe elevated expression of inflammatory genes in the hypothalamus of female rats on HFD (Fig.5). However, we observed increased hypothalamic expression of IL-l*β* and TNF-*α* (Fig.5) in LPS groups that may explain the observed hyperphagia.

In spite of the pronounced effects of LPS and HFD on caloric intake hypothalamic expression of appetite-regulating genes was not affected by HFD, and only expression of orexin was elevated by the low dose of LPS on control and HFD (Table3). Orexin stimulates food intake in rats (Sakurai et al. 1998) and acute LPS stimulus can diminish orexin expression, conveying sickness behavior (Gaykema and Goehler 2009) and reducing food intake. However, here we observed a biphasic LPS effect on orexin expression that did not correspond with the differences in energy intake. This could indicate involvement of different appetite-regulating mechanisms not only between LPS and HFD, but also depending on the dose of LPS. The lack of changes in expression patterns of other appetite-regulating genes is puzzling in light of the observed changes in food and calorie intake and resultant body mass, and might be attributed to minor, undetectable fluctuations or possible action via other pathways maintaining energy balance (Camilleri 2015).

Despite the significant body mass gain, neither HFD nor LPS significantly altered glucose tolerance and insulin sensitivity in these rats (Fig.4). This is surprising because high-fat feeding induces insulin resistance in both mice and rats (Storlien et al. 1993; Winzell and Ahren 2004) and because acute and chronic LPS exposure induces insulin resistance in mice (Cani et al. 2007) and humans (Agwunobi et al. 2000; Mehta et al. 2010). Animal models of diet-induced obesity often require long-term exposure to HFD and it can take up to year before glucose intolerance is pronounced (Winzell and Ahren 2004; Hariri and Thibault 2010). Here, we treated the rats for 5–6 weeks before the OGTTs and merely observed a tendency for increased AUC for insulin and glucose upon glucose bolus in the HFD rats (Fig.4). It is possible that the exposure time to HFD was too short for the rats to develop prominent insulin resistance and glucose intolerance. Nonetheless, neither dose of LPS affected female rat glucose metabolism (Fig.4). It is an indication that chronic intraperitoneal delivery of a low dose of LPS to female rats does not induce glucose intolerance in spite of hypothalamic inflammation (Fig.5). Glucose and insulin values 120 min after glucose administration correspond well with those previously observed in Sprague Dawley rats at the same time point during an OGTT (Hendricks and Mahoney 1972; Stark et al. 2000) or after a shorter fast of 6 h (Cacho et al. 2008), now commonly utilized before OGTTs (Andrikopoulos et al. 2008). Even though future studies should also examine incretin levels in these rats, the absence of differences in OGTT results indicate otherwise.

Indeed, species and sex of the experimental animal may play an important role in immune response and progression of development of diabetes (Pietschmann et al. 2003; Franconi et al. 2008), however Pohl et al. (Pohl et al. 2013) describe only a slight attenuation of inflammatory response to acute LPS exposure in female rats compared to male rats (Pohl et al. 2009). Here, we observed increased food intake and body mass in response to LPS in cycling female rats that has not been previously observed in male rats exposed to chronic LPS (Smith et al. 2006; Liu et al. 2010; Fischer et al. 2015) and could be attributed to sex differences. On the other hand species differences may also be important, as chronic LPS administration increased body mass in male mice (Cani et al. 2007). In the present study, we observed an immune response among female rats treated with LPS (Fig.5) that was not followed by a reduction in glucose tolerance, in spite of significant weight gain (Fig.3B). While there is no clear evidence whether females are more protected than males against development of T2D (Gale and Gillespie 2001; Logue et al. 2011), females may have a different response to inflammatory stimuli than males (Bouman et al. 2004). Additionally the administration site of LPS (subcutaneous vs. intraperitoneal) may also play an important role in conveying and responding to the inflammatory stimulus (Dantzer et al. 1998). Subcutaneous delivery of LPS induced insulin resistance in male mice (Cani et al. 2007), whereas 4-week long intraportal or intraperitoneal infusion of LPS in male rats failed to induce insulin resistance (Liu et al. 2010; Fischer et al. 2015). Future studies are needed to identify the effects of species, sex, and administration site on LPS-induced phenotype. Sprague Dawley rats have a high interindividual variation in susceptibility to diet-induced obesity and insulin resistance (Ghibaudi et al. 2002) and thus may not be the most reliable model for this type of studies. However, most of studies investigating effects of chronic LPS administration used male Sprague Dawley rats (Smith et al. 2006, 2009; Liu et al. 2010; Fischer et al. 2015), and in order to avoid strain differences we used female Sprague Dawley rats. It is, however, possible that absence of LPS and HFD effects on glucose tolerance can be attributed to the specificity of the Sprague Dawley strain and should be investigated in other, more susceptible animal models (e.g., C57 mice).

## Conclusions

Our study showed that exposing adult female Sprague Dawley rats to chronic intraperitoneal LPS infusion for 7.5 weeks increased their body mass and calorie intake, but did not affect their glucose tolerance. LPS elicited a dose-dependent response, with rats receiving highest dose of LPS gaining the most body mass. Consumption of high-fat diets not only further exacerbated the effects of LPS on body mass and energy intake, but also failed to induce detectable glucose intolerance. Interestingly, in spite of the inflammatory nature of HFD only LPS increased expression of proinflammatory genes in the hypothalamus.

## Conflict of Interest

None declared.

## References

[b1] Agwunobi AO, Reid C, Maycock P, Little RA, Carlson GL (2000). Insulin resistance and substrate utilization in human endotoxemia. J. Clin. Endocrinol. Metab.

[b2] Alexander C, Rietschel ET (2001). Bacterial lipopolysaccharides and innate immunity. J. Endotoxin Res.

[b3] Amar J, Burcelin R, Ruidavets JB, Cani PD, Fauvel J, Alessi MC (2008). Energy intake is associated with endotoxemia in apparently healthy men. Am. J. Clin. Nutr.

[b4] Andersen CL, Jensen JL, Orntoft TF (2004). Normalization of real-time quantitative reverse transcription-PCR data: a model-based variance estimation approach to identify genes suited for normalization, applied to bladder and colon cancer data sets. Cancer Res.

[b5] Andrikopoulos S, Blair AR, Deluca N, Fam BC, Proietto J (2008). Evaluating the glucose tolerance test in mice. Am. J. Physiol. Endocrinol. Metab.

[b6] Bonefeld BE, Elfving B, Wegener G (2008). Reference genes for normalization: a study of rat brain tissue. Synapse.

[b7] Bouman A, Schipper M, Heineman MJ, Faas MM (2004). Gender difference in the non-specific and specific immune response in humans. Am. J. Reprod. Immunol.

[b8] Cacho J, Sevillano J, de Castro J, Herrera E, Ramos M (2008). Validation of simple indexes to assess insulin sensitivity during pregnancy in Wistar and Sprague-Dawley rats. Am. J. Physiol. Endocrinol. Metab.

[b9] Camilleri M (2015). Peripheral mechanisms in appetite regulation. Gastroenterology.

[b10] Cani PD, Amar J, Iglesias MA, Poggi M, Knauf C, Bastelica D (2007). Metabolic endotoxemia initiates obesity and insulin resistance. Diabetes.

[b11] Cani PD, Bibiloni R, Knauf C, Neyrinck AM, Neyrinck AM, Delzenne NM (2008). Changes in gut microbiota control metabolic endotoxemia-induced inflammation in high-fat diet-induced obesity and diabetes in mice. Diabetes.

[b12] Dantzer R, Bluthé R-M, Layé S, Bret-Dibat J-L, Parnet P, Kelley KW (1998). Cytokines and sickness behavior. Ann. N. Y. Acad. Sci.

[b13] De Souza CT, Araujo EP, Bordin S, Ashimine R, Zollner RL, Boschero AC (2005). Consumption of a fat-rich diet activates a proinflammatory response and induces insulin resistance in the hypothalamus. Endocrinology.

[b14] Elfving B, Bonefeld BE, Rosenberg R, Wegener G (2008). Differential expression of synaptic vesicle proteins after repeated electroconvulsive seizures in rat frontal cortex and hippocampus. Synapse.

[b15] Fischer CW, Liebenberg N, Madsen AM, Müller HK, Lund S, Wegener G (2015). Chronic lipopolysaccharide infusion fails to induce depressive-like behaviour in adult male rats. Acta Neuropsychiatr.

[b16] Franconi F, Seghieri G, Canu S, Straface E, Campesi I, Malorni W (2008). Are the available experimental models of type 2 diabetes appropriate for a gender perspective?. Pharmacol. Res.

[b17] Gale EAM, Gillespie KM (2001). Diabetes and gender. Diabetologia.

[b18] Gaykema RPA, Goehler LE (2009). Lipopolysaccharide challenge-induced suppression of Fos in hypothalamic orexin neurons: their potential role in sickness behavior. Brain Behav. Immun.

[b19] Geary N, Asarian L, Sheahan J, Langhans W (2004). Estradiol-mediated increases in the anorexia induced by intraperitoneal injection of bacterial lipopolysaccharide in female rats. Physiol. Behav.

[b20] Ghibaudi L, Cook J, Farley C, van Heek M, Hwa JJ (2002). Fat intake affects adiposity, comorbidity factors, and energy metabolism of sprague-dawley rats. Obes. Res.

[b21] Gruys E, Toussaint MJ, Niewold TA, Koopmans SJ (2005). Acute phase reaction and acute phase proteins. J. Zhejiang Univ. Sci. B.

[b22] Hariri N, Thibault L (2010). High-fat diet-induced obesity in animal models. Nutr. Res. Rev.

[b23] Hendricks D, Mahoney A (1972). Glucose tolerance in zinc-deficient rats. J. Nutr.

[b24] Hill JO (2006). Understanding and addressing the epidemic of obesity: an energy balance perspective. Endocr. Rev.

[b25] Hotamisligil GS, Shargill NS, Spiegelman BM (1993). Adipose expression of tumor necrosis factor-alpha: direct role in obesity-linked insulin resistance. Science.

[b26] Kelly CJ, Colgan SP, Frank DN (2012). Of microbes and meals: the health consequences of dietary endotoxemia. Nutr. Clin. Pract.

[b27] Kleemann R, Zadelaar S, Kooistra T (2008). Cytokines and atherosclerosis: a comprehensive review of studies in mice. Cardiovasc. Res.

[b28] Koenig W, Sund M, Frohlich M, Fischer HG, Lowel H, Doring A (1999). C-Reactive protein, a sensitive marker of inflammation, predicts future risk of coronary heart disease in initially healthy middle-aged men: results from the MONICA (Monitoring Trends and Determinants in Cardiovascular Disease) Augsburg Cohort Study, 1984 to 1992. Circulation.

[b29] Laugerette F, Vors C, Peretti N, Michalski MC (2011). Complex links between dietary lipids, endogenous endotoxins and metabolic inflammation. Biochimie.

[b30] Lawrence CB, Brough D, Knight EM (2012). Obese mice exhibit an altered behavioural and inflammatory response to lipopolysaccharide. Dis. Model. Mech.

[b31] Liu T-T, Kao C-C, Chung C-F, Hsieh P-S (2010). Chronic hepatic inflammation induced by mild portal endotoxemia is not associated with systemic insulin resistance in rats. J. Med. Sci.

[b32] Logue J, Walker JJ, Colhoun HM, Leese GP, Lindsay RS, McKnight JA (2011). Do men develop type 2 diabetes at lower body mass indices than women?. Diabetologia.

[b33] Lu Y-C, Yeh W-C, Ohashi PS (2008). LPS/TLR4 signal transduction pathway. Cytokine.

[b34] de Luca C, Olefsky JM (2008). Inflammation and insulin resistance. FEBS Lett.

[b35] Lugarini F, Hrupka BJ, Schwartz GJ, Plata-Salaman CR, Langhans W (2005). Acute and chronic administration of immunomodulators induces anorexia in Zucker rats. Physiol. Behav.

[b36] Mehta NN, McGillicuddy FC, Anderson PD, Hinkle CC, Shah R, Pruscino L (2010). Experimental endotoxemia induces adipose inflammation and insulin resistance in humans. Diabetes.

[b37] O’Reilly B, Vander AJ, Kluger MJ (1988). Effects of chronic infusion of lipopolysaccharide on food intake and body temperature of the rat. Physiol. Behav.

[b38] Park HS, Park JY, Yu R (2005). Relationship of obesity and visceral adiposity with serum concentrations of CRP, TNF-alpha and IL-6. Diabetes Res. Clin. Pract.

[b39] Pietschmann P, Gollob E, Brosch S, Hahn P, Kudlacek S, Willheim M (2003). The effect of age and gender on cytokine production by human peripheral blood mononuclear cells and markers of bone metabolism. Exp. Gerontol.

[b40] Pohl J, Woodside B, Luheshi GN (2009). Changes in hypothalamically mediated acute-phase inflammatory responses to lipopolysaccharide in diet-induced obese rats. Endocrinology.

[b41] Pohl J, Luheshi GN, Woodside B (2013). Effect of obesity on the acute inflammatory response in pregnant and cycling female rats. J. Neuroendocrinol.

[b42] Posey KA, Clegg DJ, Printz RL, Byun J, Morton GJ, Vivekanandan-Giri A (2009). Hypothalamic proinflammatory lipid accumulation, inflammation, and insulin resistance in rats fed a high-fat diet. Am. J. Physiol. Endocrinol. Metab.

[b43] Pradhan AD, Manson JE, Rifai N, Buring JE, Ridker PM (2001). C-reactive protein, interleukin 6, and risk of developing type 2 diabetes mellitus. JAMA.

[b44] Sakurai T, Amemiya A, Ishii M, Matsuzaki I, Chemelli RM, Tanaka H (1998). Orexins and orexin receptors: a family of hypothalamic neuropeptides and G protein-coupled receptors that regulate feeding behavior. Cell.

[b45] Shi H, Kokoeva MV, Inouye K, Tzameli I, Yin H, Flier JS (2006). TLR4 links innate immunity and fatty acid-induced insulin resistance. J. Clin. Investig.

[b46] Smith BJ, Lerner MR, Bu SY, Lucas EA, Hanas JS, Lightfoot SA (2006). Systemic bone loss and induction of coronary vessel disease in a rat model of chronic inflammation. Bone.

[b47] Smith BJ, Lightfoot SA, Lerner MR, Denson KD, Morgan DL, Hanas JS (2009). Induction of cardiovascular pathology in a novel model of low-grade chronic inflammation. Cardiovasc. Pathol.

[b48] Stark A, Timar B, Madar Z (2000). Adaption of Sprague Dawley rats to long-term feeding of high fat of high fructose diets. Eur. J. Nutr.

[b49] Storlien LH, Pan DA, Kriketos AD, Baur LA (1993). High fat diet-induced insulin resistance. Ann. N. Y. Acad. Sci.

[b50] Thaler JP, Yi CX, Schur EA, Guyenet SJ, Hwang BH, Dietrich MO (2012). Obesity is associated with hypothalamic injury in rodents and humans. J. Clin. Investig.

[b51] Winzell MS, Ahren B (2004). The high-fat diet-fed mouse – a model for studying mechanisms and treatment of impaired glucose tolerance and type 2 diabetes. Diabetes.

